# Anti-fibrotic effects of pirfenidone and rapamycin in primary IPF fibroblasts and human alveolar epithelial cells

**DOI:** 10.1186/s12890-018-0626-4

**Published:** 2018-04-27

**Authors:** M. Molina-Molina, C. Machahua-Huamani, V. Vicens-Zygmunt, R. Llatjós, I. Escobar, E. Sala-Llinas, P. Luburich-Hernaiz, J. Dorca, A. Montes-Worboys

**Affiliations:** 1 Department of Pneumology, Bellvitge University Hospital, Barcelona, Spain; 2 Pneumology Research Group, IDIBELL, University of Barcelona, Barcelona, Spain; 3 Research Network in Respiratory Diseases (CIBERES), ISCIII, Madrid, Spain; 4 Department of Pathology, Bellvitge University Hospital, Barcelona, Spain; 5 Department of Thoracic Surgery, Bellvitge University Hospital, Barcelona, Spain; 6 Servei de Diagnostic per la Imatge El Prat (SDPI El Prat) Department of Radiology, Bellvitge University Hospital, Barcelona, Spain; 7 Department of Penumology, Son Espases University Hospital, Palma de Mallorca, Spain; 8 Laboratori de Pneumologia Experimental (Lab. 4126). IDIBELL, Pavelló de Govern. Campus de Bellvitge, Universitat de Barcelona, Hospital de Bellvitge, Carrer de la Feixa Llarga, 08907 L’Hospitalet de Llobregat, Barcelona, Spain

**Keywords:** Pirfenidone, Rapamycin, Idiopathic pulmonary fibrosis, Pulmonary fibrosis, Cell migration, Extracellular matrix proteins, Epithelial-mesenchymal transition

## Abstract

**Background:**

Pirfenidone, a pleiotropic anti-fibrotic treatment, has been shown to slow down disease progression of idiopathic pulmonary fibrosis (IPF), a fatal and devastating lung disease. Rapamycin, an inhibitor of fibroblast proliferation could be a potential anti-fibrotic drug to improve the effects of pirfenidone.

**Methods:**

Primary lung fibroblasts from IPF patients and human alveolar epithelial cells (A549) were treated in vitro with pirfenidone and rapamycin in the presence or absence of transforming growth factor β1 (TGF−β). Extracellular matrix protein and gene expression of markers involved in lung fibrosis (tenascin-c, fibronectin, collagen I [COL1A1], collagen III [COL3A1] and α-smooth muscle actin [α-SMA]) were analyzed. A cell migration assay in pirfenidone, rapamycin and TGF−β-containing media was performed.

**Results:**

Gene and protein expression of tenascin-c and fibronectin of fibrotic fibroblasts were reduced by pirfenidone or rapamycin treatment. Pirfenidone-rapamycin treatment did not revert the epithelial to mesenchymal transition pathway activated by TGF−β. However, the drug combination significantly abrogated fibroblast to myofibroblast transition. The inhibitory effect of pirfenidone on fibroblast migration in the scratch-wound assay was potentiated by rapamycin combination.

**Conclusions:**

These findings indicate that the combination of pirfenidone and rapamycin widen the inhibition range of fibrogenic markers and prevents fibroblast migration. These results would open a new line of research for an anti-fibrotic combination therapeutic approach.

## Background

Idiopathic pulmonary fibrosis (IPF) is the most common and deadly form of idiopathic interstitial pneumonia. The mortality from diagnosis is estimated between 3 and 5 years, and onset of the disease usually occurs in elderly adults [1]. The pathogenic mechanisms are still unclear; however, recent studies agree that the disease is the result of abnormal wound healing in response to micro-injuries of the alveolar epithelium. The activated alveolar epithelial cells cause the migration, proliferation, and activation of mesenchymal cells with the formation of myofibroblast foci [2]. The myofibroblasts secrete excessive amounts of extracellular matrix (ECM) proteins, including tenascin-c, fibronectin and collagens, with the subsequent distortion of lung homoeostasis and architecture [1, 3]. These changes in the ECM composition besides an increase in cytokines and growth factors, such as transforming growth factor (TGF)−β, contribute to IPF progression [2].

Over the past decade, growing evidence has demonstrated the clinical benefits of anti-fibrotic treatment in IPF, and two anti-fibrotic drugs are now recommended in patients in the mild-moderate stage of the disease [4]. In particular, pirfenidone (5-methyl-1-phenyl-2-[1H]-pyridone) has been shown to slow down the decline in FVC. [5, 6]. Pirfenidone is a pleiotropic molecule that inhibits TGF−β, collagen synthesis, and fibroblast proliferation, and mediates tissue repair [7–12]. The anti-fibrotic activity and safety of this novel agent has been established in lung, liver and kidney tissue [13]. Several molecular mechanisms responsible for the anti-fibrotic action of pirfenidone are still under study. The current clinical objective in IPF treatment is to inhibit disease progression with anti-fibrotic therapy [14], and the current research objective is to find an anti-fibrotic capable of completely halting or abrogate fibrosis. In this context, the in vitro anti-fibrotic effect of drug combinations, together with the safety profile of this therapeutic approach, should be explored in future clinical trials.

Rapamycin, an inhibitor of mammalian target of rapamycin (mTOR)-mediated and a potent anti-proliferative drug, was initially introduced into clinical practice to prevent transplant rejection and later to treat mTOR diseases such as lymphangioleiomiomatosis (LAM) [15, 16]. Rapamycin presents some metabolic and bioavailability differences with other analogs such as everolimus [17]. All the rapalogs are structurally similar to rapamycin differing mainly at a single position of the lactone ring (C-40) [17]. Although everolimus exhibits greater polarity than rapamycin, the bioavailability is only slightly improved and is still relatively low. There are differences in the half-life, potentially affecting the optimal dosing schedules [17]. More recently, the potent anti-fibrogenic action of rapamycin has been demonstrated in animal models of hepatic [18], renal [19], and pulmonary fibrosis [20]. The anti-fibrotic effects of rapamycin in human lung fibroblasts are mediated by a decrease in collagen synthesis (COL1A1, COL1A2, and COL3A1) [20, 21]. Thus, it has been suggested as a new anti-fibrotic pathway to explore in pulmonary fibrosis [22] and is being tested in clinical trials.

Currently, the efforts of the scientific community are focused on the search for anti-fibrotic strategies that stop the abnormal repair process that leads to fibrosis and dysfunction of the lung tissue in order to avoid the progression of this devastating disease. Within this framework, it is essential to find novel agents or combinations of anti-fibrotic therapies. In this study, we aim to elucidate the role of pirfenidone and rapamycin as a new therapeutic approach for the treatment of lung fibrosis by analyzing first their anti-fibrotic potential in vitro as a primary step.

## Methods

### Isolation of human lung fibroblasts

Adult human lung fibroblasts were obtained from lung biopsies of six different IPF patients who underwent surgical biopsy for the diagnosis of the disease (histologically confirmed usual interstitial pneumonia). The harvested lung tissue samples were maintained in DMEM high Glucose with L-Glutamine (Gibco Life Technologies) medium with HEPES (Sigma-Aldrich, St Louis, MO, USA) and insulin human transferrin and sodium selenite (ITS) (Sigma-Aldrich) until processing. Then cut into small pieces, and placed into 6 well plates (Nunc Thermo Scientific, Waltham, MA, USA) with growth medium; DMEM supplemented with 10% fetal bovine serum (FBS, Gibco Life Technologies), penicillin (100 U/ml)/streptomycin (100 μg/ml) solution (Gibco Life Technologies) and 25 μg/ml amphotericin B (Sigma-Aldrich). Cells were cultured at 37 °C in a humidified atmosphere of 5% CO_2_. Spindle-like primary fibroblasts started to grow separately from tissue samples on day 2 to 3. Outgrowth of fibroblasts took 1 to 2 weeks. Tissue samples were then removed by aspiration, and cells were allowed to reach confluence. Fibroblasts at confluence were expanded by trypsinization and passaged every 4 to 5 days at 1:4 ratio. Pulmonary fibroblasts were identified by the typical spindle morphology and immunohistochemistry; vimentin and α-smooth muscle actin (α-SMA) positive, and factor VIII and surfactant C-negative staining. Cells between passage numbers 4 and 7 were used in this study.

### Cell line culture

Bronchial-alveolar epithelial human cells (A549) were purchased from the American Type Culture Collection (ATCC CRM-CCL-185, Manassas, VA, USA) and cultured in F12 K (Gibco Life Technologies, Grand Island, NY, USA) medium supplemented with 10% FBS (Gibco Life Technologies) and penicillin (100 U/ml) / streptomycin (100 μg/ml) solution (Gibco Life Technologies) according to the manufacturer’s recommendations. Cells were maintained at 37 °C in a humidified 5% CO_2_ atmosphere.

### Cell viability assay

Cell viability was evaluated using a commercial colorimetric assay (Quick Cell Proliferation Assay Kit II. MBL, International Corporation, Woburn, MA, USA) according to the recommended protocol. Briefly, cells (5 × 10^4^/well) were cultured in a 96-well microtiter plate (Nunc Thermo Scientific) and treated with pirfenidone (Hoffmann-La Roche) (1 mg/ml), and rapamycin (Sigma-Aldrich) (1 μg/ml), and the combination of both agents in the presence of TGF−β (5 ng/ml) in a final volume of 100 μl/well of 2% FBS culture medium in triplicates for 72 h. Then, 10 μl/well of WST reagent was added and plates were incubated for 2 h at 37 °C in standard culture conditions. After shaking the plates for 1 min, the absorbance was computed at a wavelength of 450 nm in each well using a microplate reader (Thermo Scientific) with 650 nm of reference wavelength. The amount of the dye generated by activity of dehydrogenase is directly proportional to the number of living cells.

### Cell culture stimulation

Human lung primary fibroblasts and A549 cell line were cultured in 6 well plates (Nunc Thermo Scientific) in the appropriated medium with 10% FBS; when cells reached 80% confluence the medium was changed at 2% FBS. Cells were stimulated with activated TGF−β (5 ng/ml) (R&D Systems Minneapolis, MN, USA), in the presence of pirfenidone (1 mg/ml) and rapamycin (1 μg/ml) during 72 h. Besides, we stimulated cells with a combination of rapamycin and pirfenidone without TGF−β, rapamycin with TGF-β, pirfenidone with TGF−β and rapamycin, pirfenidone and TGF−β. After the incubation period, cells and supernatants were collected, separated by centrifugation and frozen for further analysis. Doses of pirfenidone and rapamycin were chosen based on previous reported studies [7, 9, 23–27] and our results from the cell viability assay.

### Western blot assay

Cells were grown in 6-well plates (Nunc) and incubated with TGF−β (5 ng/ml), pirfenidone (1 mg/ml) and rapamycin (1 μg/ml) for 72 h at 37 °C. Cells were then lysed in Radio-Immunoprecipitation Assay (RIPA) Buffer (25 mM Tris-HCl, 150 mM NaCl, 1% NP-40, 0.1% sodium deoxycholate SDS) containing 1:100 phenylmethylsulfonyl fluoride and phosphatase inhibitors (Sigma-Aldrich). The final protein concentrations were determined with a bicinchoninic acid (BCA) method (Thermo Scientific) according to the manufacturer’s specifications. Prepared samples were heated to 100 °C for 5 min; for each sample the same amount of total protein (20–30 μg) was added to a well of 4–15% mini-protean TGX precast gels polyacrylamide gel (Bio-Rad Hercules, CA, USA) and resolved by SDS-PAGE. The separated proteins were transferred to a nitrocellulose membrane (Bio-Rad). The membranes were blocked for 1 h in Tris-buffered saline (10 mM Tris-HCl pH 7.5 and 0.15 M NaCl) containing 0.1% (*v*/v) Tween 20 and 5% (*w*/*v*) bovine serum albumin (BSA) (Sigma-Aldrich), and then probed at room temperature (RT) for 1 h with primary antibodies against human tenascin-C (diluted 1:500; Abcam, Cambridge, UK #ab3970), human E-cadherin (diluted 1:2000, Abcam #ab76055), human EDA-fibronectin (diluted 1:400, Abcam #ab6328), human collagen I (diluted 1:200 Abcam #ab88147), human collagen III (diluted 1:500 Abcam #ab6310) human α-SMA (diluted 1:1000, Sigma #A5228), human α-tubulin (diluted 1:5000, Sigma Aldrich # T6199), human vinculin (diluted 1:5000, Abcam #ab129002) and human β-actin (diluted 1:5000, Sigma #A1978). Immunoreactive bands were detected with IgG horseradish peroxidase-conjugated secondary antibodies (anti-mouse diluted 1:1000; and anti-rabbit diluted 1:1000) (Dako, Glostrup, Denmark) and visualized by enhance chemiluminescence detection reagents ECL Western blotting kit (Bio-Rad) according to the manufacturer’s instructions in a luminescent image analyzer (LAS 3000 Fujifilm) and were then scanned for densitometry analysis (Multi Gauge software, Fujifilm). Results were expressed as a ratio of band density to total β-actin, vinculin or α-tubulin.

### RNA extraction and real-time polymerase chain reaction (RT-PCR)

Total RNA was isolated from cultured cells, after treatment with TGF−β, pirfenidone and rapamycin following the same protocol explained above, using the Qiagen RNeasy Mini Kit (Qiagen, Valencia, CA, USA) according to the manufacturer’s recommendations. Samples were digested with DNase I (Qiagen) to remove contaminating genomic DNA. RNA concentration and purity of each sample were measured using UV spectrophotometry. A total of 1 μg of RNA was reverse-transcribed using the iScript cDNA synthesis kit (Bio Rad) with oligo deoxythymidine and random hexamer primers. The reverse transcriptase reaction proceeded in a total volume of 20 μl in a conventional thermal cycler (Bio-Rad) at 25 °C for 5 min, followed by 30 min at 42 °C and 5 min at 85 °C. Reaction volumes of 20 μl were placed in 384-well optical reaction plates with adhesive covers (ABI Prism™ Applied Biosystems, Foster City, CA, USA) using SYBR Green PCR Master Mix and specific sequence primers (Sigma). Glyceraldehyde-3-phosphate dehydrogenase (GADPH) mRNA amplified from the same samples served as the internal control. Samples were heated to 95 °C for 10 min and then PCR amplification was achieved by 40 cycles at 95 °C for 15 s and 60 °C for 1 min using the ABI Prism 7900 (Applied Biosystems). The relative expression of each targeted gene was normalized by subtracting the corresponding housekeeping genes (β-actin, GADPH, HPRT and RNA18s) threshold cycle (Ct) value using the comparative CT method (∆∆C_t_ methods).

### Cell migration assay

Cell migration was monitored from a confluent area to an area that was mechanically denuded of cells (scratch-wound assay). Cells were grown in 6-well plates (Nunc) to a confluent monolayer and then serum-deprived for 24 h. After the medium was discarded, a scratch was created in a straight line across the cells with a p20 pipette tip. The plates were then rinsed with phosphate buffered saline (PBS) to remove the suspended cells and incubated with the specific media supplemented with TGF−β (5 ng/ml), pirfenidone (1 mg/ml), rapamycin (1 μg/ml) or the combination of the three agents. Twenty-four and 72 h after the treatment, cells were monitored and photographed under a light microscope, and the distance cover by the cells in the wound closure was analyzed with Image J software (https://imagej.nih.gov/ij/ National Institute of Health NIH, Bethesda, MD, USA). Cells treated with drug-free medium were considered as controls.

### Statistical analysis

All results are expressed as mean ± SEM of independent experiments. Statistical analysis was performed in Graph Pad Prism 5.01 (Graph Pad Software, San Diego, CA, USA). Significance is indicated as follows: *p < 0.05, **p < 0.01, ***p < 0.001. * indicates the comparison between samples treated with TGF−β and all the other conditions: TGF−β versus untreated samples (control); TGF−β versus rapamycin and pirfenidone (rapa/pirfe); and samples treated with TGF−β in combination with rapamycin (TGF−β/rapa); pirfenidone (TGF−β/pirfe) or both (TGF−β/rapa/pirfe). ^#^*p* < 0.05, ^##^*p* < 0.01, ^###^*p* < 0.001. # indicates significant differences between control versus samples treated with rapamycin and pirfenidone in absence of TGF−β.

## Results

### Pirfenidone and rapamycin are well tolerated by human lung fibroblasts and alveolar epithelial cells

**Fig. 1:**
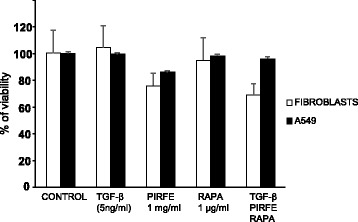
Effect of TGF−β, pirfenidone and rapamycin on the viability of lung primary fibroblasts and A549 cells. Primary lung fibroblasts and bronchial epithelial human cells (A549) were incubated with pirfenidone (1 mg/ml) and rapamycin (1 μg/ml) in addition to the combination of the two agents with TGF−β (5 ng/ml) for 72 h, viability was measured with the WST reagent. No significant cytotoxic effect was observed for any combinatory treatments. The bars represent the mean values ± SEM of viability % related to control samples in three different experiments

Cell viability was assayed with the WST reagent in fibroblasts and A549 cells cultured in 96-well plates and treated with TGF−β (5 ng/ml), pirfenidone (1 mg/ml) and rapamycin (1 μg/ml) for 72 h. The cell toxicity assay demonstrated that pirfenidone and rapamycin, in addition to the combination of the 2 agents with TGF−β (5 ng/ml), did not cause cellular death when compared to untreated cells (control) and were well tolerated for the treatment period of 72 h (Fig. 1). We then selected the doses of 1 mg/ml for pirfenidone, in accordance with the results obtained by other Groups [7, 9, 23–27], 1 μg/ml rapamycin and 5 ng/ml of TGF−β to be used in the following studies for a treatment period of 72 h.

### Pirfenidone and rapamycin treatment inhibits ECM protein expression in lung fibrotic fibroblasts

Fibroblasts were obtained from IPF patients submitted to lung biopsies for the diagnosis of interstitial disease. After the cultures were established, we stimulated the cells with TGF−β (5 ng/ml), pirfenidone (1 mg/ml) and rapamycin (1 μg/ml). Besides the treatment of rapamycin and pirfenidone without TGF−β, rapamycin alone with TGF−β, pirfenidone alone with TGF−β and the combination of rapamycin and pirfenidone with TGF−β for 72 h. Western blot and RT-PCR were performed to analyze the protein and gene expression of the different markers involved in the fibrotic process.

We observed a significant decrease in tenascin-c, and collagen III when cells were incubated with pirfenidone or rapamycin.

#### Tenascin-c

**Fig. 2:**
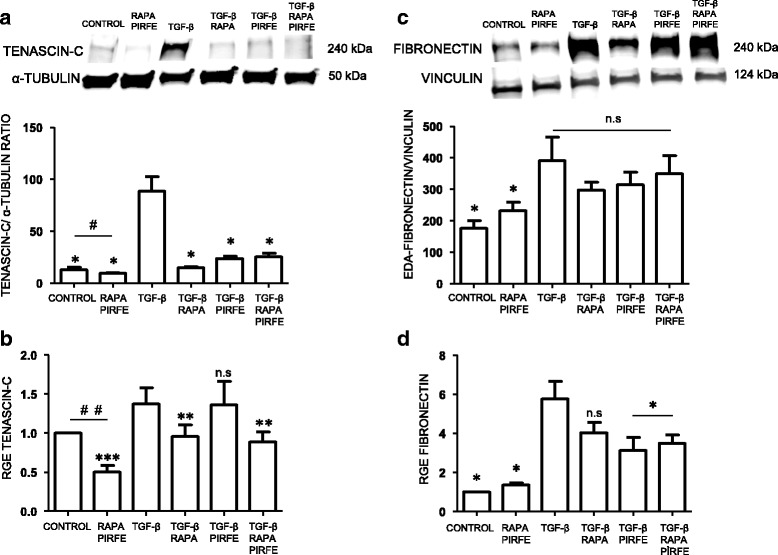
Pirfenidone and rapamycin effect in protein and gene expression of tenascin-c and fibronectin in human lung fibroblasts from IPF patients. Primary lung fibroblasts from IPF patients were treated with TGF−β (5 ng/ml), pirfenidone (1 mg/ml) and rapamycin (1 μg/ml) for 72 h. **a** The total cell lysates were subjected to immunoblot analysis for tenascin-c. The data shown are mean values ± SEM of four different experiments. **b** Results of tenascin-c transcript fold changes expressed as relative gene expression (RGE) analyzed by RT-PCR. **c** Fibronectin protein level. Density of protein bands were normalized against vinculin and are shown as ratios. **d** Fibronectin transcript fold changes expressed as relative gene expression (RGE). Charts represent mean values ± SEM of four different experiments. Levels of significance: *p < 0.05; ** *p* < 0.01; ***p < 0.001. * indicates the comparison between samples treated with TGF−β and all the other conditions: TGF−β versus untreated samples (control); rapamycin and pirfenidone (rapa/pirfe); and samples treated with TGF−β in combination with rapamycin (TGF−β/rapa); pirfenidone (TGF−β/pirfe) or both (TGF−β/rapa/pirfe). ^#^*p* < 0.05; ^##^ *p* < 0.01. # indicates the comparison between untreated cells (control) and samples treated with rapamycin and pirfenidone in the absence of TGF-β (rapa/pirfe). n.s statistically not significant

Previous studies conducted by our Group [28] and others [29] have shown a clear induction of tenascin-c in the fibrotic fibroblasts from IPF patients compared to other interstitial diseases. We wanted to test the effect of pirfenidone and rapamycin on tenascin-c in lung fibroblasts from IPF patients treated with the pro-fibrotic factor TGF−β. As shown in Fig. 2a, b, we found clear inhibition of protein and gene expression levels when cells were treated with both drugs. The response of the treatment in the presence of TGF−β were clearer in both the gene expression analysis and the protein level. However, the effect was likely mediated by rapamycin. Furthermore, the incubation with pirfenidone and rapamycin in absence of TGF−β resulted in a significant decrease of tenascin-c, as compared control cells with rapamycin-pirfenidone treatment.

#### Fibronectin

Another component of the ECM that plays an important role in the maintenance of lung homeostasis and wound healing is fibronectin (FN). Due to its implication in the development of pulmonary fibrosis and its pro-fibrotic role in IPF, we analyzed the protein and gene expression of FN in lung fibroblasts. As shown in Fig. 2c, we observed a significant increase of protein level in TGF−β stimulated fibroblasts when compared to the control. However, we found a non-significant reduction in the FN protein expression when cells were incubated with pirfenidone and rapamycin. The gene expression of fibronectin induced by TGF−β was clearly inhibited by the combination of pirfenidone and rapamycin, but the effect might likely be due to the action of pirfenidone (Fig. 2d). Moreover, when cells were stimulated with rapamycin and pirfenidone in absence of TGF−β we observed a slight increase of protein and gene expression of fibronectin but this was not statistically significant.

#### Collagen type I and III

**Fig. 3:**
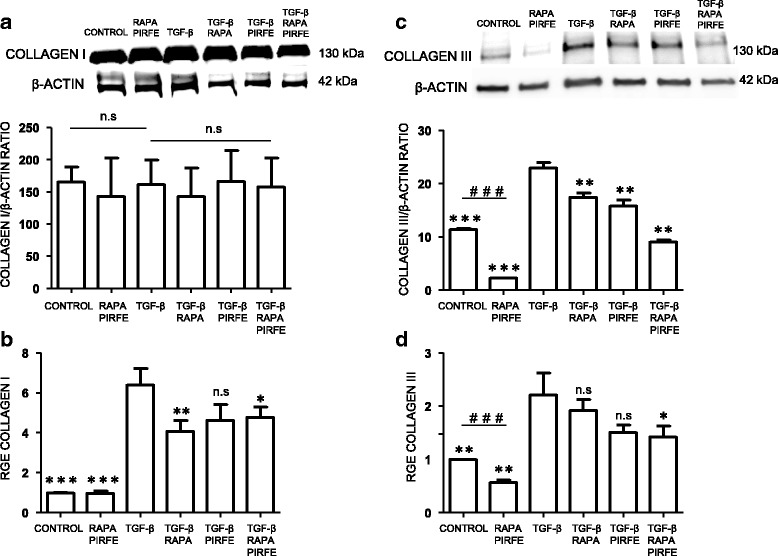
Collagen I and III gene expression are decreased by pirfenidone and rapamycin, while only the protein expression level of collagen III is inhibited by pirfenidone and rapamycin. Primary lung fibroblasts from IPF patients were treated with TGF−β (5 ng/ml), pirfenidone (1 mg/ml) and rapamycin (1 μg/ml) for 72 h. **a** Densitometric analysis expressed as mean ± SEM and a representative blot showing the unchanged effect in COL1A1 levels by pirfenidone and rapamycin. **b** COL1A1 gene expression measured by RT-PCR was decreased by pirfenidone and rapamycin in IPF fibroblasts. Bars represent mean ± SEM values of the transcript fold changes expressed by relative gene expression (RGE) of four different experiments. **c** COL3A1 protein expression decreases with the combination treatment of rapamycin and pirfenidone. Bars expressed as mean ± SEM values. Treated samples with rapamycin and pirfenidone in the absence of TGF−β show a significant decrease of the protein compare to controls. **d** COL3A1 gene expression showing a significant decrease when cells were treated with rapamycin and pirfenidone combination treatment. Levels of significance: *p < 0.05; ** *p* < 0.01; ***p < 0.001. * indicates the comparison between samples treated with TGF−β and all the other conditions: TGF−β versus untreated samples (control); rapamycin and pirfenidone (rapa/pirfe); and samples treated with TGF−β in combination with rapamycin (TGF−β/rapa); pirfenidone (TGF−β/pirfe) or both (TGF−β/rapa/pirfe). ^###^ *p* < 0.001. # indicates the comparison between untreated cells (control) and samples treated with rapamycin and pirfenidone (rapa/pirfe). n.s statistically not significant

The overexpression of collagen in fibrotic lungs has been shown to be a key factor in tissue dysfunction. Following the same protocol, we analyzed the levels of collagen type I (COL1A1) and collagen type III (COL3A1) in lung fibroblasts when they were stimulated with TGF−β, pirfenidone and rapamycin for 72 h. The combination of pirfenidone and rapamycin with or without TGF−β did not alter the high basal levels of COL1A1 protein expression in fibroblasts from IPF patients. This high level of protein expression in control samples could due to the fibroblasts´ origin from fibrotic lung areas. On the other hand, levels of COL3A1 protein were inhibited by the treatment of rapamycin and pirfenidone in the presence of TGF−β. Furthermore, the gene expression of both collagen I and III after treatment with TGF−β was abolished by pirfenidone or rapamycin, the most definitive response to the combined treatment of pirfenidone and rapamycin after TGF−β was found in collagen III (Fig. 3a, b, c, d).

### The fibroblast to myofibroblast transition is inhibited by pirfenidone and rapamycin treatment combination

**Fig. 4:**
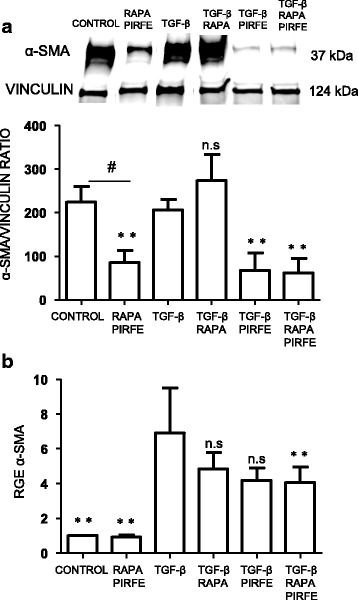
Fibroblast to myofibroblast transformation is inhibited by pirfenidone and rapamycin treatment. α-SMA marker was studied in primary lung fibroblasts from IPF patients treated with TGF−β (5 ng/ml), pirfenidone (1 mg/ml) and rapamycin (1 μg/ml) for 72 h. **a** Western blot analysis in cell lysates. Graphs represent means ± SEM of target/control ratios obtained by densitometric analysis of each experiment results. **b** The analysis of α-SMA gene expression after 72 h treatment showed differences between samples treated with TGF−β in the absence or presence of pirfenidone and rapamycin. α-SMA transcript fold changes expressed as relative gene expression (RGE) measured by RT-PCR in fibroblasts from IPF patients, mean ± SEM of four different experiments normalized using four different housekeeping genes as control (β-actin, GADPH, HPRT, and RNA18s). Levels of significance: ** *p* < 0.01; ***p < 0.001.* indicates the comparison between samples treated with TGF−β and all the other conditions: TGF−β versus untreated samples (control); rapamycin and pirfenidone (rapa/pirfe); and samples treated with TGF−β in combination with rapamycin (TGF−β/rapa); pirfenidone (TGF−β/pirfe) or both (TGF−β/rapa/pirfe). ^#^*p* < 0.05. # indicates the comparison between untreated cells (control) and samples treated with rapamycin and pirfenidone (rapa/pirfe). n.s statistically not significant

We studied the gene and protein expression of α-SMA as an indicator for fibroblast transformation into fibrotic active myofibroblasts. As expected, fibroblasts from fibrotic lungs showed a high protein expression of α-SMA, and the treatment with TGF−β increased α-SMA gene expression (Fig. 4a, b). This increase was significantly reduced with the treatment of pirfenidone and rapamycin, although pirfenidone alone had a clearer inhibitory effect. Interestingly, cells treated with both drugs in the absence of TGF−β also showed a significant decrease of α-SMA levels when compared to control samples. These results suggest that the transformation of fibroblasts to myofibroblasts in IPF fibroblasts is inhibited by pirfenidone and rapamycin, but the effect was mainly due to the action of pirfenidone.

### Pirfenidone and rapamycin treatment prevent fibroblast migration

**Fig. 5:**
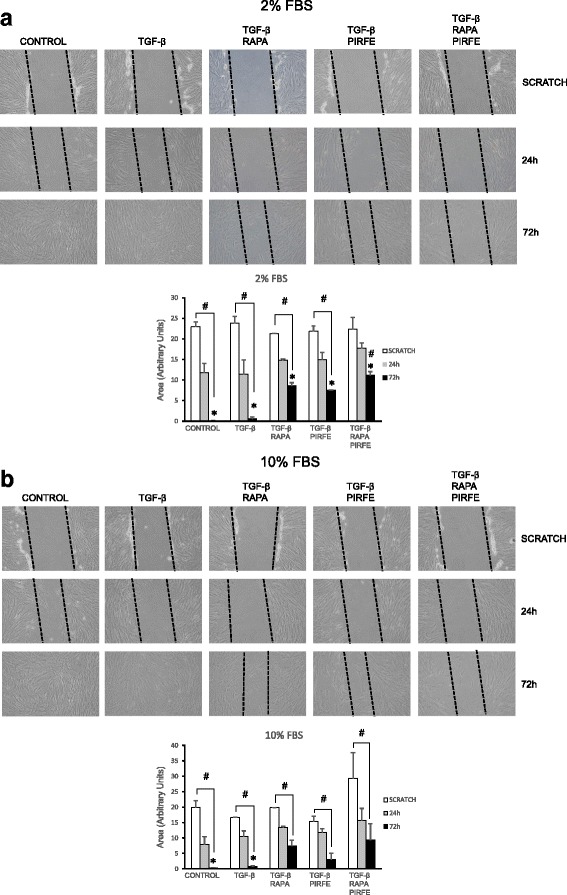
Pirfenidone and rapamycin inhibited the TGF−β-induced migration of human IPF lung fibroblasts. Cell migration assay were performed in fibroblasts from IPF patients incubated with TGF−β (5 ng/ml), rapamycin (1 μg/ml) and pirfenidone (1 mg/ml). Images show that cell migration occurred within 72 h from culture scratch in medium containing 2% (**a**) and 10% (**b**) fetal bovine serum (FBS) in the presence of TGF−β, combination of TGF−β and rapamycin, TGF−β and pirfenidone, and the combination of TGF−β, rapamycin and pirfenidone. The uncovered area has been quantified by the ImageJ Software at each time point and represented in the graphs as mean values ± SEM of three different experiments. Magnification, 100X. Levels of significance; * indicates the statistical significance between all conditions assayed at 72 h time point. # indicates the significance between scratch and 24 and 72 h time points for each treatment conditions

We performed the scratch assay with primary lung fibroblasts from IPF patients. After the scar, cells were treated with TGF−β (5 ng/ml), pirfenidone (1 mg/ml), rapamycin (1 μg/ml) and the combination of all three agents for 24 and 72 h with the medium supplemented with 2% and 10% of FBS, as explained above. The cell migration assay, analyzed with Image J software, showed a decreased number of migrated cells after a scratch wound was made across the cells and treated with pirfenidone and rapamycin or the combination of both agents when compared with untreated cells or cells stimulated with only TGF−β. In control and TGF−β treated samples, fibroblasts filled the scratch as early as 24 h after the start of incubation. In contrast, when cells were treated with pirfenidone and rapamycin, the migration took up to 72 h. The same protocol was repeated in a culture medium supplemented with 2% (Fig. 5a) and 10% (Fig. 5b) FBS to ensure that the FBS was not interfering with the migration process in control and treated cells. As shown in the graph, the same motility pattern was observed with the different concentrations of FBS assayed.

### Pirfenidone and rapamycin inhibit the synthesis of pro-fibrotic markers induced by TGF-β in alveolar epithelial cells

In order to study the effect of pirfenidone and rapamycin in lung epithelium, we conducted the same protocol explained above for primary fibroblasts with A549 cells. Culture cells were incubated with TGF−β (5 ng/ml), pirfenidone (1 mg/ml) and rapamycin (1 μg/ml) for 72 h, and after the incubation period, cells were collected and total RNA and protein were extracted.

#### Tenascin-c

**Fig. 6:**
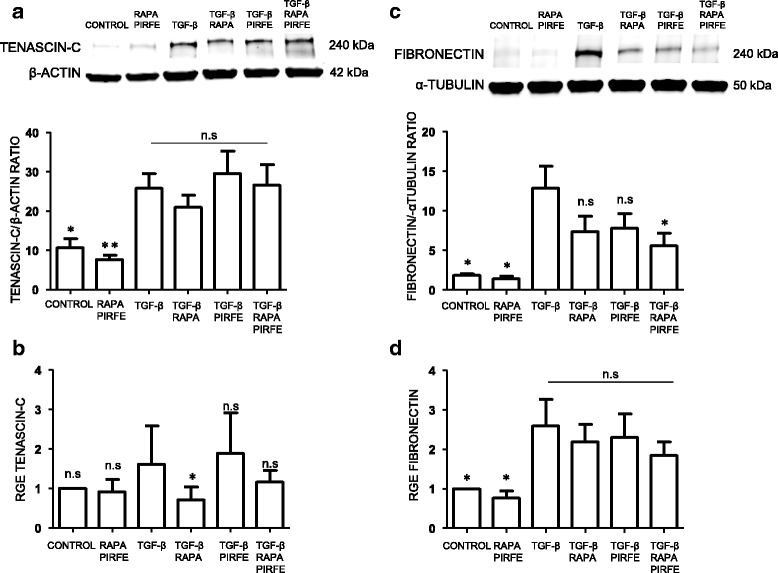
Inhibitory effect of pirfenidone and rapamycin on tenascin-c and fibronectin gene and protein expression in bronchial epithelial human cells. A549 cells were incubated with TGF−β (5 ng/ml), pirfenidone (1 mg/ml) and rapamycin (1 μg/ml). Protein and gene expression were determined after the 72 h incubation period. **a** Representative immune blot showing the expression of tenascin-c in A549 cells. The bar chart summarizes the densitometric analysis of total protein expression normalized to β-actin. **b** Analysis of tenascin-c gene expression normalized with four different housekeeping genes (β-actin, GADPH, HPRT, and RNA18s). The bars represent the mean values ± SEM of the transcript fold changes expressed as relative gene expression (RGE) in at least three different experiments. **c** Fibronectin protein expression measured in cell lysates by western blot in at least three different experiments. The bar chart represents the densitometric analysis using α-tubulin as loading control. **d** Fibronectin gene expression was normalized using four different housekeeping genes as control (β-actin, GADPH, HPRT, and RNA18s). Bars describe the mean values ± SEM of the transcript fold changes, expressed as relative gene expression (RGE) between control and samples treated with TGF−β, pirfenidone and rapamycin. Level of significance: *p < 0.05; ** *p* < 0.01. * indicates the comparison between samples treated with TGF−β and all the other conditions: TGF−β versus untreated samples (control); rapamycin and pirfenidone (rapa/pirfe); and samples treated with TGF−β in combination with rapamycin (TGF−β/rapa); pirfenidone (TGF−β/pirfe) or both (TGF−β/rapa/pirfe). n.s statistically not significant

The tenascin-c levels from A549 cells incubated with TGF−β, pirfenidone and rapamycin for 72 h were analyzed western blot and RT-PCR. We found a significant decrease of tenascin-c in cells incubated with pirfenidone and rapamycin in absence of TGF−β. However, there was only a slight decrease of tenascin-c level in cells treated with rapamycin after TGF−β, indicating the role of this drug in inhibiting the protein expression of this ECM protein. (Fig. 6a, b).

#### Fibronectin

We analyzed the protein expression of FN in A549 cells. Western blot indicated that treatment with pirfenidone and rapamycin decreased the induced FN protein levels (Fig. 6c), and treatment with both drugs alone resulted in a partial inhibition of FN. Moreover, when FN transcript was analyzed by RT-PCR, we found that pirfenidone and rapamycin combined treatment reduced the increased levels induced by TGF−β, although the difference did not reach statistical significance (Fig. 6d), supporting the results obtained in the protein expression analysis.

#### Collagen type I and III

**Fig. 7:**
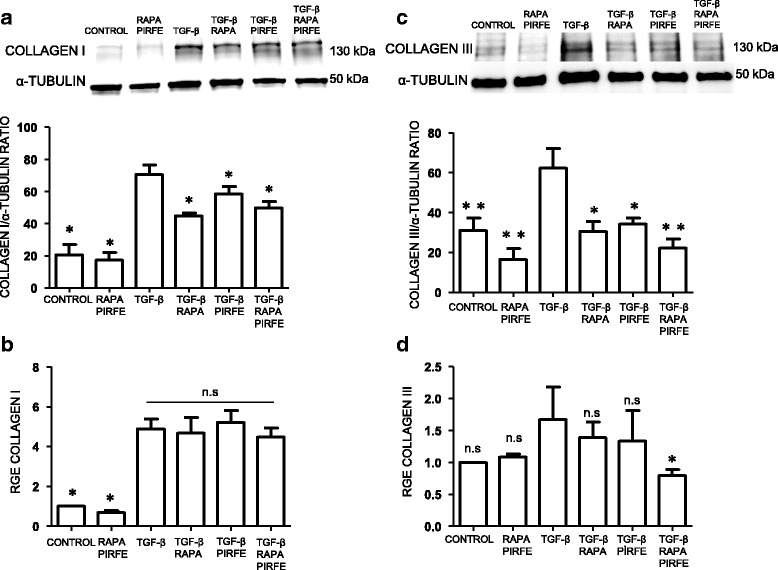
Pirfenidone and rapamycin combined treatment inhibit the expression of collagen type I and III induced by TGF−β in alveolar human epithelial cells. A549 cells were incubated with TGF−β (5 ng/ml), pirfenidone (1 mg/ml) and rapamycin (1 μg/ml) for 72 h. **a** and **c** The expression of COL1A1 and COL3A1 were measured by western blot. Representative immunoblot analysis of at least three different experiments normalized by α-tubulin. The combination of rapamycin and pirfenidone treatment inhibits the protein expression of collagen type III. Gene expression **b** and **d** were analyzed in A549 cells after the drug treatment by RT-PCR. Reported values are means ± SEM of the transcript fold changes expressed as relative gene expression (RGE) normalized by four housekeeping genes and calculated on the level of untreated (control) cells. Only collagen type III gene expression is decreased by the treatment of rapamycin and pirfenidone. Levels of significance; *p < 0.05; **p < 0.01 * indicates the comparison between samples treated with TGF−β and all the other conditions: TGF−β versus untreated samples (control); rapamycin and pirfenidone (rapa/pirfe); and samples treated with TGF−β in combination with rapamycin (TGF−β/rapa); pirfenidone (TGF−β/pirfe) or both (TGF−β/rapa/pirfe). n.s statistically not significant

We also examined the effect of pirfenidone and rapamycin treatment in the synthesis of collagen when alveolar epithelial cells were stimulated with TGF−β. We observed an increase of COL1A1 and COL3A1 protein with TGF−β that was inhibited by the presence of pirfenidone and rapamycin, but the addition of both drugs did result in a lower decrease of only the collagen type III protein (Fig. 7a, c). We then studied the gene expression of COL1A1 and COL3A1 in the same samples, and found that both genes increased their expression with TGF−β after 72 h of treatment. Pirfenidone and rapamycin combination significantly inhibited the collagen type III transcript induced by TGF−β (Fig. 7b, d).

### Pirfenidone and rapamycin treatment does not revert the epithelial to mesenchymal transition (EMT) pathway activated by TGF−β in A549 cells

To gain deeper insight into the role of the combination pirfenidone and rapamycin treatment in the inhibition of the EMT pathway activated by TGF−β, E-cadherin marker was studied in A549 cells after 72 h of treatment with TGF−β and both drugs.

#### E-cadherin

**Fig. 8:**
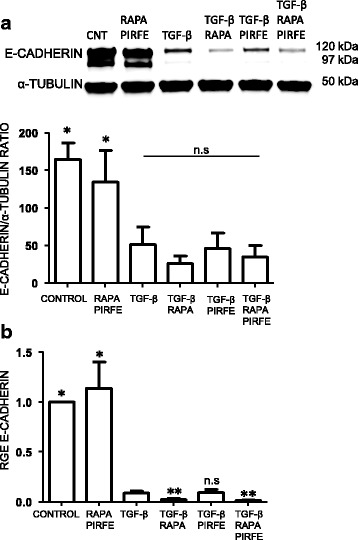
Pirfenidone and rapamycin treatment in the presence of TGF−β has no effect on EMT pathway. A549 cells were incubated with TGF−β (5 ng/ml), pirfenidone (1 mg/ml) and rapamycin (1 μg/ml) for 72 h. **a** E-cadherin protein expression measured by western blot in A549 cells. Representative blots of one out of three separate experiments. Expression of α-tubulin was used to normalize sample loading. **b** E-cadherin transcript fold changes expressed as relative gene expression (RGE) was analyzed by RT-PCR. Reported values are the mean ± SEM of the transcript fold changes expressed as relative gene expression (RGE) observed in three different experiments. Levels of significance; * *p* < 0.05; ** *p* < 0.01. * indicates the comparison between samples treated with TGF−β and all the other conditions: TGF−β versus untreated samples (control); rapamycin and pirfenidone (rapa/pirfe); and samples treated with TGF−β in combination with rapamycin (TGF−β/rapa); pirfenidone (TGF−β/pirfe) or both (TGF−β/rapa/pirfe). n.s statistically not significant

When the epithelial cell marker E-cadherin was analyzed, we observed that TGF−β inhibited the synthesis of both protein and gene expression, indicating an activation of EMT in A549 cells. Treatment with pirfenidone and rapamycin alone did not significantly affect the E-cadherin expression. However, the effect of these drugs in combination with TGF−β failed to recover the synthesis level of E-cadherin (Fig. 8a, b).

## Discussion

Pirfenidone is currently one of the primary choices for the treatment of IPF in clinical practice, however, it does not stop or cure the disease completely, suggesting that a combination with other compounds involved in different pathways could be required in order to modify the fatal outcome [14].

In this context, this study was designed to test the potential role of the combination treatment of pirfenidone with rapamycin to improve the anti-fibrotic in vitro effect. Our cytotoxic assay showed no statistically significant changes in cell viability between control and treated cells, suggesting that the used concentration of pirfenidone and rapamycin had no cytotoxic effect. Both drugs have already been used separately, with different clinical indications and satisfactory tolerance [14–16]. It has been reported that pirfenidone exhibits an inhibitory effect on a host of cell types in concentrations ranging from 0.2 to 2.0 mg/ml with no cytotoxic effect in vitro [24], and the drug is being administered orally at a daily dose of 2403 mg with tolerable degree of adverse effects in patients with pulmonary fibrosis [9]. Rapamycin, meanwhile, is already being used in cancer, transplant and LAM. An analog of rapamycin, everolimus, did not demonstrate benefit for IPF in a previous study [30]. However, everolimus and rapamycin present a different clinical profile in other diseases [31], and this indicates that choosing the correct mTOR inhibitor should be considered carefully. Rapamycin prevents and inhibits progression of ongoing pulmonary fibrosis caused by expression of TGF−α and increased epidermal growth factor receptor (EGFR) signaling [21]. Currently, rapamycin is being evaluated in a clinical trial for IPF. Furthermore, the mTOR pathway is a hallmark of aging, and recent studies have shown it to be overexpressed in the fibrotic lungs of patients with a more detrimental progression [32].

Previous studies performed by our Group have shown the importance of ECM protein expression in the fibrotic process that occurs in IPF lungs [28]. In this study, we tested the combination of pirfenidone and rapamycin treatment to abolish the synthesis of ECM proteins. We incubated the cell cultures with pirfenidone and rapamycin in absence or presence of the pro-fibrotic cytokine TGF−β. We observed different effects of pirfenidone and rapamycin when we analyzed the expression of the main proteins involved in the fibrotic process. We found that there is an inhibitory effect in the synthesis and gene expression of the ECM proteins, indicating that the treatment together is more efficient that the use of a single drug. The results indicated that rapamycin was able to inhibit the protein and gene expression of tenascin-c, and collagen I in fibroblasts and epithelial cells treated with TGF−β. On the other hand, pirfenidone succeeded in inhibiting the increase of fibronectin and α-sma in primary lung fibroblasts. These aggregated findings together demonstrated that each drug acts in different pathways and the use of both compounds would increase the anti-fibrotic range of action. Therefore, the drug combination increased the anti-fibrotic in vitro effect although a synergic effect was minimally present. Further studies are needed to better understand the role of each drug to inhibit the fibrotic process. These findings reveal the importance for a combined therapy that may result in a more efficient treatment to inhibit the ECM over-expression in the fibrotic process. The important role of both fibronectin and collagen in regulating the homeostasis of extracellular matrix and the inhibitory effect of pirfenidone in TGF−β stimulated fibroblasts has been reported [33]. Although pirfenidone has been shown to inhibit the synthesis of fibronectin and collagen in several studies, there is a discrepancy between the obtained results from protein and gene expression [34]. We demonstrate that the combination of pirfenidone with rapamycin produces more efficient results in the regulation of TGF−β-induced ECM over-expression, and highlights the importance of finding a new combinatory therapy for the treatment of pulmonary fibrosis.

Another important finding is the clear inhibition of TGF−β -induced myofibroblast transformation when fibroblasts are stimulated with pirfenidone and rapamycin, although the main effect is likely due to the action of pirfenidone. As shown in Fig. 4a, b, α-SMA was inhibited when cells were treated with both agents in the absence or presence of TGF−β, indicating a decrease in TGF−β -induced over-expression. Myofibroblasts are the last cells recruited to repair tissue damage; however, uncontrolled accumulation of this type of cell halts the healing process, resulting in the fibrotic and dysfunctional tissue. On the other hand, in agreement with other groups [35, 36], we found that pirfenidone and rapamycin failed to revert the EMT transition triggered by TGF−β in alveolar epithelial cells, as shown the studies of E-cadherin marker expression in A549 cells (Fig. 8). The discrepancy in the results we obtained in epithelial cells could be due to the use of A549 cell line. It has been reported that even when these cells are standardized in in vitro models of EMT, some factors must still be taken into account when analyzing data obtained in this cell line [7]. We would need to confirm our results in primary human type II alveolar epithelial cells from IPF patients to better understand the potential effects of pirfenidone and rapamycin on these cells.

The inhibition of cell migration by pirfenidone has been reported by other groups [9, 33] whose results are consistent with our findings in lung fibroblasts stimulated with TGF−β and then treated with pirfenidone and rapamycin. Furthermore, when cells were stimulated with both drugs simultaneously, migration was inhibited up to the last time point assayed (72 h). These results support the role of both combined agents to avoid the uncontrolled migration of fibroblasts in the injured tissue. In vivo experimental studies would be required to support the beneficial anti-fibrotic effect of pirfenidone and rapamycin combination.

## Conclusions

In summary, the present results open a new line of research into the potential of pirfenidone and rapamycin combination as a better anti-fibrotic approach and future treatment strategies.

## Abbreviations

BCA: Bicinchonic acid
ECM: Extracellular matrix protein
EGFR: Epidermal growth factor receptor signaling
ELISA: Enzyme-linked immunosorbent assay
EMT: Epithelial to mesenchymal transition
FBS: Fetal bovine serum
FN: Fibronectin
FVC: Forced vital capacity
GADPH: Glyceraldehyde-3-phosphate dehydrogrenase
HPRT: Hypoxanthine-guanine phosphoribosyltransferase
IPF: Idiopathic Pulmonary Fibrosis
ITS: Insulin human transferrin and sodium selenite
LAM: lymphangioleiomiomatosis
mTOR: Mammalian target of rapamycin
PBS: Phosphate buffered saline
RIPA: Radio immunoprecipitation assay
RT-PCR: Real-time polymerase chain reaction
TGF: Transforming growth factor
α-SMA: α-Smooth muscle actin
